# Audit of Psychosocial and Palliative Care Support for Children Having Allogeneic Stem Cell Transplants at the New Zealand National Allogeneic Transplant Centre

**DOI:** 10.3390/children8050356

**Published:** 2021-04-29

**Authors:** Amanda M. Evans, Hiran Thabrew, Bruce Arroll, Nyree Cole, Ross Drake

**Affiliations:** 1 Paediatric Palliative Care Service, Starship Children’s Health, Auckland and Mary Potter Hospice, Wellington 6242, New Zealand; 2 Consult Liaison Psychiatry Team, Starship Children’s Health and University of Auckland, Auckland 1010, New Zealand; h.thabrew@auckland.ac.nz; 3 Department of General Practice and Primary Health Care, University of Auckland, Auckland 1010, New Zealand; bruce.arroll@auckland.ac.nz; 4 Oncology and Haematogy Service, Birmingham Women’s and Children’s, Birmingham B15 2TG, UK; nyree.cole@nhs.net; 5 Paediatric Palliative Care and Pain Service, Starship Children’s Health, Auckland 1023, New Zealand; rossD@adhb.govt.nz

**Keywords:** pediatric palliative care, stem cell transplants, psychosocial support

## Abstract

Psychosocial and palliative care support during stem cell transplants (SCT) is known to improve outcomes. Aim: evaluate the support provided to children and families at the New Zealand National Allogeneic Stem Cell Transplant unit (NATC). Method: the psychosocial and palliative care support for children who received SCT between December 2012 and April 2018 was audited. Results: of the 101 children who received SCT, 97% were reviewed by the social work team (SW) and 82% by the psychiatric consult liaison team (CLT) at least once during their illness. However, pre-transplant psychological assessment only occurred in 16%, and during the SCT admission, only 55% received SW support, and 67% received CLT support. Eight out of eighty-five families (9%) were offered support for siblings. Eight of the sixteen children who died were referred for pediatric palliative care (PPC) with all supported and half the families who experienced a death (*n* = 8; 50%) received bereavement follow up. Conclusion: although the majority received some social work and psychological support, auditing against the standards suggests the consistency of involvement could be improved. Referrals for PPC were inadequate and largely for end-of-life phase. Sibling support, in particular donor siblings, had insufficient psychological assessment and support. Key recommendations are provided to address this underperformance.

## 1. Introduction

A stem cell transplant (SCT) is an intensive, potentially life-saving treatment that is offered to children and adults with hematological, oncological, metabolic, and immunological conditions [1]. It is widely acknowledged that a SCT is physically and psychologically very challenging [1,2,3,4,5,6,7,8,9,10,11,12,13,14], with physical symptoms ranging in severity from mild to life threatening [1]. The median admission length for a child is six weeks, and once discharged, a long period of recovery (often beyond a year) is necessary before they are able to attend school and enjoy normal childhood activities [4,15]. The mortality rate has decreased over time due to enhanced tissue typing, more sophisticated supportive care, and early recognition and treatment of opportunistic infections. However, morbidity is burdensome, difficult to predict, and linked with increased suffering [16,17,18,19,20].

The psychological challenges for children and families has been referred to as a “dark time” [21], with anxiety, depression, and post-traumatic stress disorder (PTSD) being relatively common [8,12,13,22,23]. Support received during the transplant admission is directly related to psychosocial outcomes post-transplant [24,25,26,27,28,29]. In children, psychological effects from a transplant may be long lasting, and can make it difficult for children to emotionally re-integrate back into their normal life and return to school [4,14,30,31]. However, evidence indicates children can adapt and return to their pre-transplant quality of life within 2 years, given the right psychosocial and palliative care [32] support during transplantation [8,22,33].

Parents of children undergoing SCT also experience distress [34,35] and can have persistent trauma-related anxiety and depression [3,34,36,37,38,39,40,41,42], which can adversely affect the child [42,43].

Pre-transplant family functioning is an important component of coping during SCT and is important to assess [44,45,46,47,48]. This assessment can identify protective and social risk factors and/or pre-existing mental health issues which would require a tailored approach by the consult liaison psychiatric team (CLT) and social work (SW) [49,50].

The effect on the wider family is evident in siblings who have a greater risk of low self-esteem, anxiety, PTSD, behavior problems, and future maladaptive coping strategies [43,51,52,53,54,55,56,57]. Balancing these negative outcomes is the potential for emotional maturity and increased empathy. Sibling donors face the additional emotional burden associated with the practical aspect of donation and, ultimately, the success or failure of the SCT. This speaks to the importance of having CLT/SW participation in the care of the child and family [58].

Pediatric palliative care (PPC) has a role in supporting children and their families going forward for SCT. SCT is a complicated medical procedure, which can cause ongoing severe morbidity. A key domain of PPC care, which has a focus on reduction of symptom burden, facilitation of effective communication, and discussion on goals of care, hopes, wishes, and fears can all serve to support quality of life, whilst living with morbidity. In addition, culminates in supporting the child at the end-of-life and management of harm associated with unsuccessful SCT.

A randomized control trial performed in adults showed that patients who received palliative care during SCT had decreased anxiety, depression, and post-traumatic stress disorder post SCT [27], with effects continuing for up to 6 months in comparison to those not receiving palliative care [28]. For children, PPC has been shown to reduce the time in intensive care and is associated with fewer interventions at the end-of-life [59,60,61].

Additionally, when a child dies during or after an SCT, parents experience more distress during bereavement, in comparison to those parents who have had a child die from cancer; this is increased further when the child dies in the pediatric intensive care unit (PICU) [62,63]. For all of these reasons, many pediatric transplant units have integrated PPC into clinical teams [32].

## 2. Aims

This audit of the psychosocial and palliative care support being offered to children and their families at NATC aimed to determine if current practice met minimum standards and, from the findings, provide recommendations for improvement.

## 3. Methods

### 3.1. The Setting of the Study

The New Zealand National Allogeneic Transplant Centre (NATC) in Auckland is the only center performing pediatric allogeneic SCT in New Zealand. Psychosocial support for both inpatients and outpatients is provided by two social workers, two play specialists, and an adolescent and youth health (AYA) nurse specialist embedded within the oncology team. Additional psychological support is provided by the pediatric CLT; a multi-disciplinary team of psychiatrists, psychologists, psychotherapists, and nurse specialists. A dedicated specialist PPC Service for the hospital supports children and their families with serious illness, cancer, and non-cancer conditions throughout the disease trajectory and provides care both in hospital and in the community.

Cultural support and advocacy is undertaken by He Kamaka Waiora (Māori health) and Tautai Fakataha Service (Pacific Support) workers.

External volunteer services/organizations include hospital grandparents, the Child Cancer Foundation (CCF), CANTEEN (an organization geared toward the needs of adolescents with cancer and their siblings), and Immune Deficiency New Zealand.

Currently the NATC does not have official policies on psychosocial care, however, there is an expectation that all children undergoing SCT and their families receive SW, play specialist and CLT support. The decisions regarding involvement of PPC, cultural, spiritual or other support services are clinical and based on the child, their illness and/or family circumstances.

### 3.2. The Standards

There are currently no specific psychosocial and palliative care guidelines for children having SCT. The American Academy of Pediatrics (2015) published recommendations for psychosocial assessment and management, family/sibling care, spiritual care, and palliative support during pediatric oncology treatment and these were modified for use in this audit by changing the words ‘cancer treatment’ to ‘SCT treatment’. They are expected minimal standards and felt to be the most appropriate as children having SCT receive high dose chemotherapy during conditioning, and have similar needs to children receiving oncology treatment. The New Zealand context also requires an understanding of the cultural support being offered to families of Māori ethnicity [64,65]. The final adapted standards and how each standard was evaluated in practice is provided in Appendix B Table A2.

The lead author (AE) conducted a retrospective review of inpatient and outpatient electronic medical records of children who had an allogeneic SCT from December 2012 to April 2018. Information was gathered from the date of diagnosis to a year post SCT and included a review of mortality data and palliative care documentation post SCT up to the end date of the audit collection period. The date of each psychosocial encounter was used to determine if it took place pre-transplant, post-transplant, or following the child’s death if they died.

The mental health records and medical notes from regional (referring) hospitals were not accessible due to being stored on separate, restricted electronic databases. Moreover, not available were the notes from play specialists, volunteer organization support workers, teachers, AYA nurse specialists, and pastoral care staff, as these were not routinely included into the medical file.

Quantitative data, including demographics and audit findings, were extracted from the clinical notes into an electronic database and analyzed using Microsoft Excel^®^ software. Descriptive statistics were used to summarize the extent to which the modified standards were being met using a gold standard (100%) denominator for each standard [66].

Social–economic risk factors was assessed using Standard 3 [49]. These risk factors were (i) low income (when social work assessment specified a ‘low income’ job or no income); (ii) low or no employment (one working adult in a two-parent family or a single parent was unemployed); (iii) the receipt of state-funded housing. “Out of Auckland” was included as an additional social risk factor due to the financial and social cost associated with travelling and isolation from being away from family, friends, local community, and support networks. Parental education level is a known social risk factor; however, this data was not available, as it was not routinely collected as part of the children’s demographic record and social work assessment. The reviewed factors were felt to be adequate to determine social risk as they had been reported to be a higher priority than parental education in a UK study looking at social risk and health outcomes [67].

Locality approval for the audit was granted by the Auckland Research Committee Auckland District Health Board (no.7917). The study was assessed as being low-risk by the Health and Disability Ethics Committee (HDEC); it did not require a full ethics review.

Sponsorship for 6 months of research time, incidental, and administration costs were received from the charitable organization, Starship Foundation, as part of a clinical/research fellowship. This audit was part of a larger concurrent project with articles yet to be published.

## 4. Results

The demographic details of the one hundred and one children who had a total of 109 SCT’s, 8 children having had a second transplant are presented in Table 1.

The results of the psychosocial standards which demonstrate the psychosocial and palliative care support given to the children having SCT is demonstrated in Table 2.

### 4.1. Standard One: Children and Young People Having SCT and Their Families Should Receive a Psychological Assessment

Sixty-eight families (67%) had a psychological assessment during their illness. Only 16% (*n* = 11; six as inpatients and five as outpatients) received the “gold standard” of a formal pre-transplant psychological assessment. The majority of the remainder (*n* = 23; 23%) had CLT support initiated during conditioning; for seven children, this was the only time of contact. Five children and families were seen after conditioning had started.

### 4.2. Standard Two: Children and Young People Having SCT and Their Families Should Have Access to Psychological Support and Interventions throughout Their SCT Trajectory

The majority of children and families (*n* = 81; 82%) received some form of CLT support during the course of their illness. Two thirds (*n* = 68) were seen during the SCT admission and five children were seen following discharge. Around half of those who received support (*n* = 52) had two or more contacts.

### 4.3. Standard Three: Children and Young People Having SCT and Their Families Should Receive a Social Work Assessment, Which Includes a Financial Assessment

Almost all children and families (*n* = 98, 97%) received SW input, with 55% (*n* = 56) receiving this during SCT admission. Forty-four percent were seen twice or more during the admission. Of the group who had input, 63 (62%) families had a documented formal, structured assessment for social and financial risk factors.

One-third (*n* = 33) had known socioeconomic risk factors detailed in Table 3 with low or no employment (41%), low income (33%), and single parent (31%) being the most prominent, while 10% of families lived in state funded housing. Thirty-eight families had no structured assessment, and noticeably more had no documentation of social risk factors, especially related to housing and condition of the house the family were living in.

Importantly, for the 45 families where SW was not involved during SCT admission, a third (*n* = 17) had documented financial risk factors and a further 18 of these lived out of Auckland.

### 4.4. Standard Four: Siblings of Children Having SCT Should Be Provided with Appropriate Supportive Services

Of the 101 children undergoing SCT, there were 160 siblings, 29 of whom were donors. Only a minority (*n* = 14; 9%) of siblings, including four sibling donors, received documented referral for emotional support from either nursing staff, CLT, SW or palliative care.

Overall, there were 28 bereaved siblings in the cohort from 14 families. Only the children from four families (29%) were offered support. Notably, only one of three bone marrow donor siblings of a child that subsequently died was offered psychological support.

### 4.5. Standard Five: Children and Young People Having SCT and Their Families Should Receive Developmentally Appropriate i.e., Pediatric Palliative Care at the End of Their Life

Sixteen children (16%) died during the audit period; seven due to transplant-related complications and nine related to complications of the disease (most commonly malignant relapse). There were 11 referrals to PPC. Only half of those who died (*n* = 8) had a referral to PPC prior to death. Two of the nine who died from disease and one who died of complications of the SCT had referral to PPC. PPC supported all families who were referred to them.

The median time from referral to death was 37.5 days (range 2–98, Interquartile range 54.5 days). The three children who remained alive were supported by PPC during specific points of their illness; one with multiple morbidities going into SCT, one who was critically unwell in PICU and one child at high risk of relapse going forward for a second SCT.

### 4.6. Standard Six: All Families Should Receive Bereavement Follow up after the Child’s Death

Following a child’s death, eight families (50%) received bereavement follow-up. PPC followed up three of these families and five were by the pediatric intensive care staff as per PICU policy. There was no difference in the rate of follow-up for families living in Auckland (*n* = 4) and out of the Auckland area (*n* = 4).

### 4.7. Standard Seven: All children of Māori Ethnicity Should Have Access to Cultural *Support*

Nineteen study participants were of Māori ethnicity. Cultural support services were involved in the care of 63% (*n* = 12) these children and families during treatment. All those who had documented referrals were seen.

### 4.8. Standard Eight: All Families Should Have a Spiritual Assessment

During treatment, sixteen families (16%) had a spiritual assessment completed by nursing, SW, CLT, or PPC staff. Only one child received documented pastoral support as an inpatient. Twelve families (12%) identified as having religious beliefs.

## 5. Discussion

This is the first time a SCT unit was benchmarked against standards identifying psychosocial and palliative support with the findings, a mixed report for the current state of psychosocial care for children and families attending the NATC. None of the audit standards were fully achieved, although access to social work and psychological support was reasonable.

### 5.1. Overall Access to Psychosocial Support

Access to psychosocial support is close to recommended standards with 82% of children and their families utilizing CLT services and 98% receiving SW input at some stage during treatment. There are no directly comparable studies of psychosocial support in similar SCT units but the findings are similar to psychosocial support provided by U.S. oncology services where over 90% of programs had SW and child life specialists providing care to children with cancer. Fewer programs had access to psychologists (60%), neuropsychologists (31%), or psychiatrists (19%) [68,69].

The high level of CLT and SW engagement was not maintained during SCT admission, with support reduced for both CLT (68%) and SW (56%), and only 52% seeing CLT and 44% seeing SW twice or more. It could be argued that this is insufficient for a high stress setting where duration of stay averages 6 weeks. However, it may also be the case that it is difficult to provide adequate emotional support during periods of critical physical illness.

Equally concerning was the finding that 33% of the unsupported families had documented social risk factors and other families received regular CLT and SW input during the SCT admission. This suggests an ad-hoc approach to service delivery and the need for improved consistency and equity of care. There may be many reasons for this but it is likely an issue in resourcing and a need to focus on embedding more psychosocial support within the NATC or improving funding of CLT services.

It is well known that having a child with a chronic illness or requiring intensive treatment, such as an SCT, increases financial strain, which in turn increases social risk [70,71]. Bilodeau (2017) has previously reported a more than doubling of financial issues for children’s families from pre-chemotherapy (15%) to post-chemotherapy (33%). This was found to be due to factors such as loss of income, loss of a job, and travelling long distances [72]. This may, in part, explain the relatively high burden of socio-economic risk factors identified in families at the NATC. Sixty percent (*n* = 61) received chemotherapy prior to SCT and just over half (*n* = 52) of children and families lived distant to the treating center.

### 5.2. Pre-Transplant Psychological Assessment

Only eleven families undergoing SCT received a pre-transplant psychology assessment; well below the 20 to 67% rates reported in previous studies of SCT services [73,74]. Assessments were mostly undertaken when children were inpatients, with around 30% seen during or after conditioning. The timing was less than ideal for a number of reasons. Firstly, psychological assessment is a key part of the work up towards SCT and vital to ensure there is a full understanding of the process and therefore contributes to informed consent [75,76,77,78,79,80]. Second, caregiver stress is known to peak from the start of conditioning through to engraftment in line with children’s level of wellness and vulnerability [22], which could unduly influence evaluations of family capacity. Finally, there may not be adequate time to initiate psychological support before a potential crisis, which is particularly relevant when parent mental health or addiction issues are identified [81].

The reasons for the lack of pre-transplant assessment were beyond the scope of this audit. However, addressing this shortfall is necessary and may be assisted by improving care pathways, viewing psychosocial evaluation as an essential pre-transplant assessment, introducing children and families to the psychosocial team prior to admission, utilizing telemedicine, and embedding and funding a robust psychosocial team within the NATC unit.

### 5.3. Sibling Support

A small number (*n* = 14; 9%) of siblings of patients undergoing SCT were offered psychological support. This included five siblings who had their sibling die, and only one of the three siblings who donated stem cells and had their sibling die. These findings are not ideal given the known psychological impact of SCT on donor and non-donor siblings. Healthy non-donor siblings have been shown to feel isolated and at increased risk of PTSD, anxiety and depression [54,55] while the psychological outcome for donor siblings is linked to the success of the SCT. Successful transplants see donor siblings have higher levels of self-esteem, mastery, happiness, and life satisfaction, but in the event of an unsuccessful transplant and death of a sibling, donors can experience self-blame, guilt, and thoughts of personal failure [82,83,84]. This emphasis on results of the SCT is risky for the sibling donor especially as SCT can have an unpredictable mortality and morbidity risk.

Routine pre-transplant psychological assessment and post-transplant follow-up of donors has been recommended by numerous clinicians involved with psychosocial care in SCT as well as requests by donors themselves [10,57,58,85]. This is not the practice in the NATC. The donor will have an independent medical assessment by a pediatrician with request for CLT involvement only if psychological issues are identified. The reasons for the lack of donor support are not always as obvious. The family who lives distant to the unit, the family’s entire focus being on the child going forward to transplant, or desire to protect the donor child from as many traumatic events as possible, to the less obvious lack of clinician awareness to the potential impact on the sibling, can all be potential explanations.

The absence of formal support does not mean there is not an opportunity for informal support of siblings. For example, the play therapist working with siblings while they play as they wait for the patient to be clinically reviewed to foster the sense of inclusion and visibility [86].

More research is required to explore the barriers, and to determine what is required to improve clinician knowledge around sibling support.

### 5.4. Cultural and Spiritual Support

New Zealand has a strong bicultural awareness with Te Tiriti o Waitangi (The Treaty of Waitangi) signed in 1840, recognized as New Zealand’s founding document. It provides a broad statement of principles upon which the nation and government was built and it is common to refer to the intention, spirit and principles of the Treaty. The principles are not considered part of NZ domestic law, but are referred to in several Acts of Parliament.

In the hospital system, this should translate to integrating Tikanga Māori (the rituals and practice that promotes Māori physical, emotional, spiritual, and whānau/family wellbeing) into the care of Māori patients. He Kamaka Waiora is the service that provides cultural support and assists Tikanga for Māori patients and their whānau when they access health services. This includes coordination of whānau accommodation, providing social, cultural, and advocacy services, and working closely with clinicians and staff to ensure that services are responsive to the needs of the patient. This service is accessed by referral and all referred children were seen.

There are other cultural support services within the hospital for other ethnic groups including the Tautai Fakataha Service (Pacific Support) and Asian Support Services, which will be important to look at in the future.

Spiritual assessment is being recognized as an important aspect of oncology care [87,88,89]. Children often experience loneliness, loss of wholeness, and suffering during treatment. Discussions around spirituality have the potential to help bring meaning to what the child and their family are enduring and to provide hope and comfort [88,90]. Spirituality can offer strength to some in times of increased distress, particularly at the end-of-life [91,92,93].

However, it was only documented as being addressed in a minority of children and families (*n* = 16; 16%) at the NATC. Conversations on spirituality and ‘meaning making’ are not the domain of any one discipline although can be seen as a role of the nondenominational pastoral care team. Although pastoral care services exist for the NATC, they are a general service available across the wider hospitals (adult and pediatric) during working hours, and are not an integral part of either the oncology or PPC services. Spiritual discussions mostly occur with nursing staff and social workers.

The minimal engagement in spiritual discussions may reflect the absence of an assessment tool at NATC, a lack of training in this area, and confidence to hold such discussions. It is also possible that informal conversations were held and not documented.

### 5.5. Palliative Care

A minority of children (16 of 101; 16%) died during or shortly after SCT. The PPC service only received referrals for eight children, primarily in the context of post-SCT malignant relapse when no further curative options were available. This would indicate the PPC service being seen as and used for end-of-life care provision, which is a small albeit intense part of the wider care provided.

Interestingly, three additional children had referrals and remained alive; PPC’s role in these cases were primarily additional emotional support, help with pain management, practical support (providing food vouchers for example), sibling support, and helping with communication between clinicians and family when their child was critically unwell.

There is existing evidence that PPC would benefit not only those at risk of mortality associated with SCT, but also those children who survive with morbidity particularly in symptom (physical and emotional) management, care co-ordination, spiritual care, care of the wider family including siblings, and communication [32,36,88,94,95,96]. Some centers have developed PPC support in the SCT as part of the package of care. St Jude’s Children’s Hospital offers universal screening consults by PPC for every child having a SCT and Dana Farber Cancer Institute and Boston Children’s Hospital have ‘triggers’ such as second SCT, organ dysfunction, high-risk disease, and active disease to prompt referral for a consult [32]. Using a similar trigger system at NATC would have meant more referrals being made, meaning more children being supported by PPC whether they survive or are at end-of-life.

### 5.6. Bereavement Care

Only half of eligible families received bereavement follow-up after the death of their child. There was no difference in the numbers living in Auckland and out of region, indicating that location of domicile is not the whole reason for deficiency in engagement.

The psychosocial standards recommend bereavement support is routine practice. Yet, the findings at NATC were similar to the results of a survey of cancer units exploring bereavement follow-up, which found a lack of formalized bereavement care and no policies outlining best care [97].

Starship Children’s Health has a single, part-time, specialized bereavement worker who co-ordinates bereavement care. The pediatric and neonatal ICU’s have dedicated nursing hours to allow phone follow up of families that had a child or baby die in their respective units. The identification of need for additional bereavement support leads to advice on where this can be provided. The PPC service provides bereavement care as part of their usual practice.

Bereavement support is also offered by charitable organizations such as the Child Cancer Foundation, CanTeen, Skylight, Grief Center, and Sands New Zealand (pregnancy, baby, and infant loss support) in different regions around New Zealand. This support is unlikely to have been captured by the data collected. There may have been individual support provided through these organizations that have not been captured by the data collected.

## 6. Limitations

The main limitation of this audit was the retrospective collation of data, which relied on documentation in the medical record as a reflection of the psychosocial support provided. Sibling support could only be identified by ‘an offer of referral’ rather than as a formal referral, as they did not have a separate medical record. Therefore, the findings are likely to be an underestimate of care, especially as community mental health, play specialist, volunteer, external support organization, and out-of-region service activity were not likely to have been recorded in the NATC medical files. Similarly, the unavailability of data prevented an appraisal of the quality of support requiring a proxy of ‘contacts’ and ‘number of contacts’ to reflect support. Further research would be required to determine the nature of psychological and palliative care support.

Finally, the findings of a single institution study may not be generalizable to other institutions.

## 7. Recommendations

The audit findings have resulted in the following recommendations being made to the NATC:

1. Development of a service policy with minimum standards (as per the audit) detailed in Appendix A (Table A1), and care pathways for delivering coordinated psychological, social, cultural, and spiritual care.
2. The service policy is to include education of staff on expectations for psychosocial and palliative care delivery when a child is admitted for an SCT.
3. Encourage improved documentation of psychosocial support within the clinical record including disciplines not routinely documenting contacts (including external organizations). This will help with future auditing.
4. To introduce ‘triggers’ for referral to PPC to better capture the children and families that would benefit from specialist PPC input not just children at the end-of-life.
5. All bereaved families to be contacted at a defined time after the death of their child and offered bereavement follow-up regardless of the location of domicile.
6. Undertake a family satisfaction survey for bereavement follow-up and care.
7. Enhance psychosocial capacity through increased funding for psychological, child psychiatry, social work, palliative care, and bereavement care services to:
  - Ensure psychosocial pre-assessment of the child and their family to better identify those at higher social and economic risk.
  - Improve support of children and families during SCT.
  - Advance support of donor siblings throughout the SCT process.
  - Maintain high quality palliative and bereavement care support.
8. Consider use of telemedicine for psychosocial pre-assessment for children and families for those who live outside of the Auckland region or in Auckland but distant to the treatment center.
9. Build in regular 3-yearly psychosocial and palliative care audit using the standards to ensure continued improvement of psychosocial care.

## 8. Conclusions

Although children and families going through SCT at the NATC are receiving psychosocial and palliative care support, there are areas of deficiency. Most children received psychosocial support early in the course of their illness but this reduced over the time of their admission despite a significant minority having identified risk factors. Siblings and, in particular, donor siblings, do not receive adequate support.

Pleasingly, cultural support was available to all children of Māori ethnicity with one hundred percent being seen. This cultural element hopefully helped provide a sense of wellbeing to Māori children and whānau.

Unfortunately, this was not apparent in other areas of significance; spiritual care was sporadic while referral for PPC and bereavement support was inadequate.

The minimally altered psychosocial standards have allowed recommendations for change in the provision of psychosocial and palliative care at the NATC and are likely to be a useful evaluation tool for similar settings internationally.

## Figures and Tables

**Table 1:** Characteristics of participants and stem cell transplants.

Participants	Number (%)
Number of children requiring a stem cell transplant (SCT) (December 2012–April 2018) Number of SCT’s (8 requiring a second SCT)	101 109
**Age (years)**	
<1	11 (10%)
1–2	10 (10%)
2–4	28 (28%)
5–11	32 (32%)
>12	20 (20%)
**Gender**	
Male	39 (39%)
Female	62 (61%)
**Ethnicity**	
Asian	7 (7%)
European	60 (60%)
Māori	19 (19%)
Pacific	15 (15%)
**Location**	
Auckland	49 (49%)
Out of Auckland (regional)	52 (52%)
**Stem Cell Transplants**	**N = 109** (amount of stem cell transplants performed)
**Conditioning**	
Myeloablative	89 (82%)
Non-myeloablative	19 (17%)
No conditioning	1 (1%)
Total body irradiation (in addition to chemotherapy conditioning)	28 (26%)
**Stem cell source**	
Bone marrow	68 (63%)
Cord	30 (28%)
Peripheral blood stem cells	11 (10%)
**Donor**	
Haploidentical	10 (9%)
Matched unrelated donor	69 (63%)
Sibling	30 (28%)
**Purpose of SCT**	**N = 109** (amount of stem cell transplants)
Oncological disease	65 (60%)
Hematological Disease	15 (14%)
Immunological Disease	19 (17%)
Metabolic disorder	8 (7%)
Neurological disease	2 (2%)

**Table 2:** Results of the Modified Psychosocial Standards.

Standard Number	Standard	Number (%) Meeting the Standard Out of N = 101
1.	Children and young people having SCT and their families should receive a psychological assessment	68 (67%)
	Gold standard being pre-transplant period.	11 (16%)
2.	Children and young people having SCT and their families should have access to psychological support and interventions throughout their SCT trajectory.	81 (82%)
3.	Children and young people having SCT and their families should have social work support.	98 (97%)
	All families should have a financial assessment.	63 (62%)
4.	Subsample:	
	Families with siblings	85 (84%)
	Number of siblings	N = 160
	Siblings of children having SCT should be provided with appropriate support services	14 (9%)
	Sibling donors	29 (18%)
	Sibling donors should be provided with appropriate psychological support	4 (14%)
5.	Subsample:	
	Children who died	16 (16%)
	All children should receive developmentally appropriate palliative care at the end of their life.	8 (50%)
6.	Subsample:	
	Children who died	16 (16%)
	All families should receive bereavement follow-up after the child’s death.	8 (50%)
7.	Subsample:	
	Number of Māori families	19 (19%)
	All children of Māori ethnicity should have access to cultural support.	12 (63%)
8.	All families should have a spiritual assessment	17 (17%)

**Table 3:** Social–economic risk factors documented per family.

Social Risk Factors	Yes	No	Not Documented
Low income	33 (33%)	31 (31%)	37 (37%)
Single parent family	31 (31%)	66 (66%)	4 (4%)
Low/no employment	41 (41%)	25 (25%)	35 (35%)
State-funded housing	9 (9%)	44 (44%)	48 (48%)
Condition of house—damp/cold/overcrowded	9 (9%)	24 (24%)	68 (67%)
Out of Auckland	52 (51%)	49 (49%)	0

## Data Availability

The data presented in this study are available on request from the corresponding author. The data are not publicly available due to permissions not being obtained by the institution where the research was conducted.

## Appendix A

**Table A1:** Recommended SCT standards for the National Allogeneic Transplant Centre.

Standards
One: all children and families having SCT require a psychological pre-assessment, which is preferably performed as an outpatient unless there are exceptional circumstances. For those with previous psychosocial support, going forward for SCT should be seen as a separate event and re-referral for SCT related assessment performed.
Two: children and young people having SCT and their family members should have access to psychological support and interventions throughout their SCT trajectory.
Three: all families with children who are having a SCT require social and financial assessment prior to transplant. If this has occurred previously, it should be reassessed pre-SCT as a separate event.
Four: (a) all siblings of children having SCT should be assessed and provided additional psychosocial support if required. (b) All sibling donors require psychological pre-assessment and psychosocial follow up at least within the first-year post SCT.
Five: pediatric palliative care shall be provided for all children at the end of their life. Pediatric palliative care referral should be considered in those children experiencing complications or ongoing morbidity that can affect their quality of life.
Six: all families have documented bereavement follow-up after the child’s death.
Seven: all children of Māori ethnicity have a referral to cultural support. If this has been denied previously, it should be re-discussed going into SCT as a separate event.
Eight: all families have a spiritual assessment regardless of religious denomination. Pastoral care should be considered as part of the psychosocial care team within NATC.

## Appendix B

**Table A2:** How the standards were evaluated.

Standard Number	Standards	Adapted Standards	How Was This Evaluated?
1.	PSS1 and PSS6: youths with cancer and families should have psychological assessment [44,98].	All (100%) children and young people having SCT and their families should receive a psychological assessment. The gold standard being ‘all children have a pre-transplant assessment’.	How many were seen before SCT admission? How many received formal pre-transplant assessments (Components should include: parental mental health assessment, child mental health assessment, assess coping strategies, family functioning, resilience, and protective factors. Consideration of other stressors)? [78] Was there documented referral to CLT? (Y/N)
2.	PSS4: youths with cancer and their family members should have access to psychosocial support and interventions throughout the cancer trajectory [99]	All (100%) children and young people having SCT and their families should have access to psychological support and interventions throughout their SCT trajectory.	If a referral has been actioned to CLT this was considered ‘access’ as CLT will do an initial assessment and often give the family phone numbers to call for further support as well as planned reviews. Were they seen during their illness by CLT? (Y/N) Were they seen during their admission when having a SCT? (Y/N) Were they seen more than once? (Y/N)
3.	PSS5: youths with cancer and families should have social/financial assessment [44,49].	All (100%) children and young people having SCT and their families should receive a social work (SW) assessment, which includes a financial assessment.	How many had a documented SW review before the SCT admission? How many had a documented financial assessment (Financial assessment includes documentation of employment status, income, marital status and housing) [49]?
4.	PSS10: siblings of children with cancer should be provided with appropriate supportive services [100].	All (100%) siblings of children and young people having SCT should be provided with appropriate supportive services. All sibling donors should be provided with appropriate psychological support.	How many families received documented offers of referral to a service that can support the siblings? How many sibling donors had an offer of psychological support? How many families with bereaved siblings had documented offers of support?
5.	PSS13: youths and families should receive developmentally appropriate palliative care at the end of their life [101].	All (100%) children and young people having SCT and their families should receive developmentally appropriate (i.e., pediatric) palliative care at the end of their life.	Of those who died, how many received care from the PPC team? When was the referral made before their death?
6.	PSS14: psychosocial care should be provided after a child’s death [102].	All (100%) families should receive bereavement follow-up after the child’s death.	Was there documentation of bereavement follow-up? (Y/N)
7.	New standard included for New Zealand cultural context.	All (100%) children, young people, and families of Māori ethnicity should have access to cultural support [64].	Was documented cultural support provided by Māori Health?
8.	PSS15: all families should have spiritual assessment [87].	All (100%) children, young people, and families should have spiritual assessment [87].	Had documented discussions about spirituality occurred? Was there documented pastoral care service involvement? As part of demographic data, has religion been identified?

Psychosocial standards (PSS) for children undergoing cancer treatment and their families and the adapted standards used for this audit. Note: This criteria is taken from the current SIPAT [78] guidelines in psychosocial assessment.

## References

[1] Ellis M.J., Patel U.D. (2006). Hematopoietic Stem-Cell Transplantation. N. Engl. J. Med..

[2] Promotion of Comfort through Early Palliative Care Consultation for Children and Adolescents Undergoing HSCT Feasibility of Implementation and Evaluation of a Proposed Practice Change. https://archive.hshsl.umaryland.edu/bitstream/handle/10713/1722/Lafond%20Capstone%20Project%20July%202%202012%20completed%20paper.pdf?sequence=1.

[3] Forinder U., Claesson L., Szybek K., Norberg A.L. (2015). Exploring the Content of Post-Traumatic Stress Symptoms among Parents after Paediatric Stem Cell Transplant. PLoS ONE.

[4] Forinder U., Posse E. (2008). ‘A life on hold’: Adolescents’ experiences of stem cell transplantation in a long-term perspective. J. Child Health Care.

[5] Felder-Puig R., Di Gallo A., Waldenmair M., Norden P., Winter A., Gadner H., Topf R. (2006). Health-related quality of life of pediatric patients receiving allogeneic stem cell or bone marrow transplantation: Results of a longitudinal, multi-center study. Bone Marrow Transplant..

[6] Parsons S.K., Shih M.-C., Duhamel K.N., Ostroff J., Mayer D.K., Austin J., Martini D.R., Williams S.E., Mee L., Sexson S. (2005). Maternal Perspectives on Children’s Health-Related Quality of Life During the First Year after Pediatric Hematopoietic Stem Cell Transplant. J. Pediatr. Psychol..

[7] Lafond D.A., Kelly K.P., Hinds P.S., Sill A., Michael M. (2015). Establishing Feasibility of Early Palliative Care Consultation in Pediatric Hematopoietic Stem Cell Transplantation. J. Pediatr. Oncol. Nurs..

[8] Barrera M., Atenafu E. (2008). Cognitive, educational, psychosocial adjustment and quality of life of children who survive hematopoietic SCT and their siblings. Bone Marrow Transplant..

[9] Phipps S., Brown R.T. (2006). Psychosocial and Behavioral Issues in Stem Cell Transplantation. Comprehensive Handbook of Childhood Cancer and Sickle Cell Disease: A Biopsychosocial Approach.

[10] Phipps S., Wiener L.S. (2015). Psychosococial issues for transplant patients and donors. Pediatric Psycho-Oncology: A Quick Reference on the Psychosocial Dimensions of Cancer.

[11] Phipps S., Dunavant M., A Garvie P., Lensing S., Rai S.N. (2002). Acute health-related quality of life in children undergoing stem cell transplant: I. Descriptive outcomes. Bone Marrow Transplant..

[12] Felder-Puig R., Peters C., Matthes-Martin S., Lamche M., Felsberger C., Gadner H., Topf R. (1999). Psychosocial adjustment of pediatric patients after allogeneic stem cell transplantation. Bone Marrow Transplant..

[13] Chang G., Ma S.J.R., Recklitis C., Syrjala K., Patel S.K., Harris L., Ms A.M.R., Ms H.T., Parsons S.K. (2011). Children’s psychological distress during pediatric HSCT: Parent and child perspectives. Pediatr. Blood Cancer.

[14] Clarke S.-A., Eiser C., Skinner R. (2008). Health-related quality of life in survivors of BMT for paediatric malignancy: A systematic review of the literature. Bone Marrow Transplant..

[15] Gordon R., Thompson C., Eckstein G. (2009). A Costing Study of Blood and Marrow Transplantation Services in NSW: Final Report.

[16] Pidala J., Kurland B., Chai X., Majhail N., Weisdorf D.J., Pavletic S., Cutler C., Jacobsohn D., Palmer J., Arai S. (2011). Patient-reported quality of life is associated with severity of chronic graft-versus-host disease as measured by NIH criteria: Report on baseline data from the Chronic GVHD Consortium. Blood.

[17] Duncan C.N., Majhail N.S., Brazauskas R., Wang Z., Cahn J.-Y., Frangoul H.A., Hayashi R.J., Hsu J.W., Kamble R.T., Kasow K.A. (2015). Long-Term Survival and Late Effects among One-Year Survivors of Second Allogeneic Hematopoietic Cell Transplantation for Relapsed Acute Leukemia and Myelodysplastic Syndromes. Biol. Blood Marrow Transplant..

[18] Baker J.N., Hinds P.S., Spunt S.L., Barfield R.C., Allen C., Powell B.C., Anderson L.H., Kane J.R. (2008). Integration of Palliative Care Practices into the Ongoing Care of Children with Cancer: Individualized Care Planning and Coordination. Pediatr. Clin. N. Am..

[19] Fraser C.J., Bhatia S., Ness K., Carter A., Francisco L., Arora M., Parker P., Forman S., Weisdorf D., Gurney J.G. (2006). Impact of chronic graft-versus-host disease on the health status of hematopoietic cell transplantation survivors: A report from the Bone Marrow Transplant Survivor Study. Blood.

[20] Zinter M.S., Holubkov R., Steurer M.A., Dvorak C.C., Duncan C.N., Sapru A., Tamburro R.F., McQuillen P.S., Pollack M.M. (2018). Pediatric Hematopoietic Cell Transplant Patients Who Survive Critical Illness Frequently Have Significant but Recoverable Decline in Functional Status. Biol. Blood Marrow Transplant..

[21] Breitwieser C.L., Vaughn L.M. (2014). “A Day in My life” Photography Project. J. Pediatr. Oncol. Nurs..

[22] Phipps S., Dunavant M., Lensing S., Rai S.N. (2005). Psychosocial Predictors of Distress in Parents of Children Undergoing Stem Cell or Bone Marrow Transplantation. J. Pediatr. Psychol..

[23] Roeland E., Mitchell W., Elia G., Thornberry K., Herman H., Cain J., Atayee R., Bardwell W., Gunten C.F.V. (2010). Symptom control in SCT—MDT palliative care team approach. Part 2: Psychosocial concerns. J. Support. Oncol..

[24] Jacobsen P.B., Sadler I.J., Booth-Jones M., Soety E., Weitzner M.A., Fields K.K. (2002). Predictors of posttraumatic stress disorder symptomatology following bone marrow transplantation for cancer. J. Consult. Clin. Psychol..

[25] Esser P., Kuba K., Scherwath A., Schirmer L., Schulz-Kindermann F., Dinkel A., Balck F., Koch U., Kröger N., Götze H. (2016). Posttraumatic stress disorder symptomatology in the course of allogeneic HSCT: A prospective study. J. Cancer Surviv..

[26] El-Jawahri A., Traeger L., Kuzmuk K., Eusebio J., VanDusen H.B., Keenan T., Shin J., Gallagher E.R., Greer J.A., Pirl W.F. (2015). Prognostic understanding, quality of life and mood in patients undergoing hematopoietic stem cell transplantation. Bone Marrow Transplant..

[27] El-Jawahri A., Leblanc T., VanDusen H., Traeger L., Greer J.A., Pirl W.F., Jackson V.A., Telles J., Rhodes A., Spitzer T.R. (2016). Effect of Inpatient Palliative Care on Quality of Life 2 Weeks after Hematopoietic Stem Cell Transplantation. JAMA.

[28] El-Jawahri A., Temel J.S. (2017). Palliative Care Integration in Hematopoietic Stem-Cell Transplantation: The Need for Additional Research. J. Oncol. Pract..

[29] Kronenberger W.G., Carter B.D., Stewart J., Morrow C., Martin K., Gowan D., Sender L. (1996). Psychological adjustment of children in the pretransplant phase of bone marrow transplantation: Relationships with parent distress, parent stress, and child coping. J. Clin. Psychol. Med. Settings.

[30] An H., Lee S. (2019). Difficulty in returning to school among adolescent leukemia survivors: A qualitative descriptive study. Eur. J. Oncol. Nurs..

[31] Pillay B., Lee S.J., Katona L., Burney S., Avery S. (2014). Psychosocial factors associated with quality of life in allogeneic stem cell transplant patients prior to transplant. Psycho Oncol..

[32] Levine D.R., Baker J.N., Wolfe J., Lehmann L.E., Ullrich C. (2017). Strange Bedfellows No More: How Integrated Stem-Cell Transplantation and Palliative Care Programs Can Together Improve End-of-Life Care. J. Oncol. Pract..

[33] Keogh F., O’Riordan J., McNamara C., Duggan C., McCann S.R. (1998). Psychosocial adaptation of patients and families following bone marrow transplantation: A prospective, longitudinal study. Bone Marrow Transplant..

[34] Yoo J., Halley M.C., Lown E.A., Yank V., Ort K., Cowan M.J., Dorsey M.J., Smith H., Iyengar S., Scalchunes C. (2019). Supporting caregivers during hematopoietic cell transplantation for children with primary immunodeficiency disorders. J. Allergy Clin. Immunol..

[35] Terrin N., Rodday A.M., Tighiouart H., Chang G., Parsons S.K. (2012). Parental emotional functioning declines with occurrence of clinical complications in pediatric hematopoietic stem cell transplant. Support. Care Cancer.

[36] Mayer D.K., Tighiouart H., Terrin N., Stewart S., Peterson E., Jeruss S., Parsons S.K. (2009). A Brief Report of Caregiver Needs and Resource Utilization During Pediatric Hematopoietic Stem Cell Transplantation. J. Pediatr. Oncol. Nurs..

[37] Forinder U. (2004). Bone Marrow Transplantation from a Parental Perspective. J. Child Health Care.

[38] DuHamel K.N., Manne S., Nereo N., Ostroff J., Martini R., Parsons S., Williams S., Mee L., Sexson S., Wu L. (2004). Cognitive Processing among Mothers of Children Undergoing Bone Marrow/Stem Cell Transplantation. Psychosom. Med..

[39] Manne S., Duhamel K., Ostroff J., Parsons S., Martini D.R., Williams S.E., Mee L., Sexson S., Austin J., Difede J. (2004). Anxiety, depressive, and posttraumatic stress disorders among mothers of pediatric survivors of hematopoietic stem cell transplantation. Pediatrics.

[40] Vrijmoet-Wiersma C.M.J., Egeler R.M., Koopman H.M., Norberg A.L., Grootenhuis M.A. (2009). Parental stress before, during, and after pediatric stem cell transplantation: A review article. Support. Care Cancer.

[41] Vrijmoet-Wiersma C.M.J., Egeler R.M., Koopman H.M., Bresters D., Norberg A.L., A Grootenhuis M. (2009). Parental stress and perceived vulnerability at 5 and 10 years after pediatric SCT. Bone Marrow Transplant..

[42] Çoban Ö.G., Adanır A.S., Özatalay E. (2017). Post-traumatic stress disorder and health-related quality of life in the siblings of the pediatric bone marrow transplantation survivors and post-traumatic stress disorder in their mothers. Pediatr. Transplant..

[43] Packman W., Weber S., Wallace J., Bugescu N. (2010). Psychological effects of hematopoietic SCT on pediatric patients, siblings and parents: A review. Bone Marrow Transplant..

[44] Kazak A.E., Abrams A.N., Banks J., Christofferson J., DiDonato S., Grootenhuis M.A., Kabour M., Madan-Swain A., Patel S.K., Zadeh S. (2015). Psychosocial Assessment as a Standard of Care in Pediatric Cancer. Pediatr. Blood Cancer.

[45] Tillery R., Joffe N.E., Mara C.A., Davies S.M., Pai A.L. (2018). Longitudinal examination of family efficacy following pediatric stem cell transplant. Psycho-Oncology.

[46] Kupst M.J., Bingen K. (2006). Stress and Coping in the Pediatric Cancer Experience. Comprehensive Handbook of Childhood Cancer and Sickle Cell Disease: A Biopsychosocial Approach.

[47] Kupst M.J., Natta M.B., Schulman J.L., Lavigne J.V., Das L., Richardson C.C. (1995). Family Coping with Pediatric Leukemia: Ten Years after Treatment. J. Pediatr. Psychol..

[48] Jobe-Shields L., Alderfer M.A., Barrera M., Vannatta K., Currier J.M., Phipps S. (2009). Parental Depression and Family Environment Predict Distress in Children Prior to Stem Cell Transplantation. J. Dev. Behav. Pediatr..

[49] Pelletier W., Bona K. (2015). Assessment of Financial Burden as a Standard of Care in Pediatric Oncology. Pediatr. Blood Cancer.

[50] Kearney J.A., Salley C.G., Muriel A.C. (2015). Standards of Psychosocial Care for Parents of Children with Cancer. Pediatr. Blood Cancer.

[51] Wilkins K.L., Woodgate R.L. (2007). Supporting Siblings Through the Pediatric Bone Marrow Transplant Trajectory. Cancer Nurs..

[52] Pot-Mees C.C., Zeitlin H. (1987). Psychosocial Consequences of Bone Marrow Transplantation in Children. J. Psychosoc. Oncol..

[53] Packman W., Horsley H., Davies B., Kramer R. (2006). Sibling Bereavement and Continuing Bonds. Death Stud..

[54] Packman W.L. (1999). Review Psychosocial impact of pediatric BMT on siblings. Bone Marrow Transplant..

[55] Packman W.L., Crittenden M.R., Fischer J.B., Schaeffer E., Bongar B., Cowan M.J. (1998). Siblings’ Perceptions of the Bone Marrow Transplantation Process Siblings’ Perceptions of the Bone Marrow Transplantation Process. J. Psychosoc. Oncol..

[56] Packman W.L., Beck V.L., Vanzutphen K.H., Long J.K., Spengler G. (2003). The Human Figure Drawing with Donor and Nondonor Siblings of Pediatric Bone Marrow Transplant Patients. Art Ther..

[57] Pentz R.D., Alderfer M.A., Pelletier W., Stegenga K., Haight A.E., Hendershot K.A., Dixon M., Fairclough D., Hinds P. (2014). Unmet Needs of Siblings of Pediatric Stem Cell Transplant Recipients. Pediatrics.

[58] Wiener L., Hoag J.A., Pelletier W., Shah N.N., Shaw B.E., Pulsipher M.A., Bruce J., Bader P., Willasch A.M., Dalissier A. (2019). Transplant center practices for psychosocial assessment and management of pediatric hematopoietic stem cell donors. Bone Marrow Transplant..

[59] Ullrich C., Dussel V., Hilden J., Sheaffer J., Lehmann L., Wolfe J. (2010). End-of-Life Experience of Children Undergoing Hematopoietic Stem-Cell Transplantation: Parent and Provider Perspectives and Patterns of Care. J. Pain Symptom Manag..

[60] Ullrich C., Lehmann L., London W.B., Guo D., Sridharan M., Wolfe J. (2016). Pediatric Palliative Care and End of Life Outcomes for Pediatric Hematopoietic Stem Cell Transplant Recipients Who Do Not Survive. Biol. Blood Marrow Transplant..

[61] Snaman J.M., Kaye E.C., Lu J.J., Sykes A., Baker J.N. (2017). Palliative Care Involvement Is Associated with Less Intensive End-of-Life Care in Adolescent and Young Adult Oncology Patients. J. Palliat. Med..

[62] Jalmsell L., Onelöv E., Steineck G., Henter J.-I., Kreicbergs U., Onel E. (2010). Hematopoietic stem cell transplantation in children with cancer and the risk of long-term psychological morbidity in the bereaved parents. Bone Marrow Transplant..

[63] Drew D., Goodenough B., Maurice L., Foreman T., Willis L. (2005). Parental grieving after a child dies from cancer: Is stress from stem cell transplant a factor?. Int. J. Palliat. Nurs..

[64] Walker T., Signal L., Russell M., Smiler K., Tuhiwai-Ruru R. (2008). The road we travel: Maori experience of cancer. N. Z. Med. J..

[65] Egan R., McKechnie R., Jobson J., Herbison P., Richards R., Richards R. (2013). Perspectives on Psychosocial and Spiritual Cancer Support Services in New Zealand. J. Psychosoc. Oncol..

[66] Shaw M. (2002). Principles for Best Practice in Clinical Audit Principles for Best Practice in Clinical Audit. Nurs. Stand..

[67] Blank L., Baxter S., Messina J., Fairbrother H., Goyder L., Chilcott J. (2012). Summary Review of the Factors Relating to Risk of Children Experiencing Social and Emotional Difficulties and Cognitive Difficulties. https://www.nice.org.uk/guidance/ph40/documents/social-and-emotional-wellbeing-early-years-review-32.

[68] Scialla M.A., Canter K.S., Chen F.F., Kolb E.A., Sandler E., Wiener L., Kazak A.E. (2017). Implementing the psychosocial standards in pediatric cancer: Current staffing and services available. Pediatr. Blood Cancer.

[69] Scialla M.A., Canter K.S., Chen F.F., Kolb E.A., Sandler E., Wiener L., Kazak A.E. (2017). Delivery of care consistent with the psychosocial standards in pediatric cancer: Current practices in the United States. Pediatr. Blood Cancer.

[70] Heath J.A., Lintuuran R.M., Rigguto G., Tikotlian N., McCarthy M. (2006). CHILDHOOD CANCER: Its Impact and Financial Costs for Australian Families. Pediatr. Hematol. Oncol..

[71] Creswell P.D., Wisk L.E., Litzelman K., Allchin A., Witt W.P. (2014). Parental depressive symptoms and childhood cancer: The importance of financial difficulties. Support. Care Cancer.

[72] Bilodeau M., Madeline B., Al-Sayegh H., Wolfe J., Bona K. (2018). Household material hardship in families of children post-chemotherapy. Pediatr. Blood Cancer.

[73] Sherman A.C., Simonton S., Latif U., Nieder M.L., Adams R.H., Mehta P. (2004). Psychosocial supportive care for children receiving stem cell transplantation: Practice patterns across centers. Bone Marrow Transplant..

[74] Mc Farland D., Gorman E., Kim S., Rothwell A., Saunders P., Tindle S., De La Vega-Diaz I., Steinberg A. (2015). Psychiatric evaluations prior to stem cell transplant—A survey of National Marrow Donor Programs. Psycho-Oncology.

[75] Grover S., Sarkar S. (2012). Liver Transplant—Psychiatric and Psychosocial Aspects. J. Clin. Exp. Hepatol..

[76] Fung E., Shaw R.J. (2008). Pediatric Transplant Rating Instrument—A scale for the pretransplant psychiatric evaluation of pediatric organ transplant recipients. Pediatr. Transplant..

[77] Freischlag K.W., Chen V., Nagaraj S.K., Chua A.N., Chen D., Wigfall D.R., Foreman J.W., Gbadegesin R., Vikraman D., Chambers E.T. (2019). Psychosocial Assessment of Candidates for Transplantation (PACT) Score Identifies High Risk Patients in Pediatric Renal Transplantation. Front. Pediatr..

[78] Maldonado J.R., Dubois H.C., David E.E., Sher Y., Lolak S., Dyal J., Witten D. (2012). The Stanford Integrated Psychosocial Assessment for Transplantation (SIPAT): A New Tool for the Psychosocial Evaluation of Pre-Transplant Candidates. J. Psychosom. Res..

[79] Pai A.L., Swain A.M., Chen F.F., Hwang W.-T., Vega G., Carlson O., Ortiz F.A., Canter K., Joffe N., Kolb E.A. (2019). Screening for Family Psychosocial Risk in Pediatric Hematopoietic Stem Cell Transplantation with the Psychosocial Assessment Tool. Biol. Blood Marrow Transplant..

[80] Cousino M.K., Schumacher K.R., Rea K.E., Eder S., Zamberlan M., Jordan J., Fredericks E.M. (2018). Psychosocial functioning in pediatric heart transplant recipients and their families. Pediatr. Transplant..

[81] Manne S., Mee L., Bartell A., Sands S., Kashy D.A. (2016). A randomized clinical trial of a parent-focused social-cognitive processing intervention for caregivers of children undergoing hematopoetic stem cell transplantation. J. Consult. Clin. Psychol..

[82] Switzer G., Dew M., Magistro C., Goycoolea J., Twillman R., Alter C., Simmons R. (1998). The effects of bereavement on adult sibling bone marrow donors’ psychological well-being and reactions to donation. Bone Marrow Transplant..

[83] D’Auria J.P., Fitzgerald T.M., Presler C.M., Kasow K.A. (2015). Through the Eyes of Young Sibling Donors: The Hematopoietic Stem Cell Donation Experience. J. Pediatr. Nurs..

[84] MacLeod K.D., Whitsett S.F., Mash E.J., Pelletier W. (2003). Pediatric Sibling Donors of Successful and Unsuccessful Hematopoietic Stem Cell Transplants (HSCT): A Qualitative Study of Their Psychosocial Experience. J. Pediatr. Psychol..

[85] Pillay B., Lee S.J., Katona L., De Bono S., Warren N., Fletcher J., Burney S. (2012). The psychosocial impact of haematopoietic SCT on sibling donors. Bone Marrow Transplant..

[86] Gaab E.M. (2002). Perceptions of Life and Death in Paediatric Palliative Care. https://www.semanticscholar.org/paper/Perceptions-of-Life-and-Death-in-Paediatric-Care%3A-Gaab/b77868fe772a09b5d33a1f3f67329d536ff5bcd3.

[87] Robert R., Stavinoha P., Jones B.L., Robinson J., Larson K., Hicklen R., Smith B., Perko K., Koch K., Findley S. (2019). Spiritual assessment and spiritual care offerings as a standard of care in pediatric oncology: A recommendation informed by a systematic review of the literature. Pediatr. Blood Cancer.

[88] Mack J.W., Evan E., Duncan J., Wolfe J. (2015). Chapter 70—Palliative Care in Pediatric Oncology.

[89] Peteet J.R., Balboni M.J. (2013). Spirituality and religion in oncology. CA Cancer J. Clin..

[90] Leget C., Macleod R., Block L. (2018). Spirituality in Pall Care. Textbook of Palliative Care.

[91] Robinson M., Thiel M., Backus M., Meyer E.C. (2011). Matters of Spirituality at the End of Life in the Pediatric Intensive Care Unit (abstract). Pediatrics.

[92] Meert K.L., Thurston C.S., Briller S.H. (2005). The spiritual needs of parents at the time of their child’s death in the pediatric intensive care unit and during bereavement: A qualitative study. Pediatr. Crit. Care Med..

[93] Foster T.L., Bell C.J., Gilmer M.J. (2012). Symptom Management of Spiritual Suffering in Pediatric Palliative Care. J. Hosp. Palliat. Nurs..

[94] Wood C., Rowe A., D’Souza A., Speedy L., Adler J., Carter J.M. (2016). The Palliative Care Service and the Stem Cell Transplant Unit—A Happy Marriage. Biol. Blood Marrow Transplant..

[95] Baker J.N., Barfield R., Hinds P.S., Kane J.R. (2007). A Process to Facilitate Decision Making in Pediatric Stem Cell Transplantation: The Individualized Care Planning and Coordination Model. Biol. Blood Marrow Transplant..

[96] Zhukovsky D.S., Herzog C.E., Kaur G., Palmer J.L., Bruera E. (2009). The Impact of Palliative Care Consultation on Symptom Assessment, Communication Needs, and Palliative Interventions in Pediatric Patients with Cancer. J. Palliat. Med..

[97] Wiener L., Rosenberg A.R., Lichtenthal W.G., Tager J., Weaver M.S. (2018). Personalized and yet standardized: An informed approach to the integration of bereavement care in pediatric oncology settings. Palliat. Support. Care.

[98] Kearney J.A., Byrne M.W. (2014). Understanding parental behavior in pediatric palliative care: Attachment theory as a paradigm. Palliat. Support. Care.

[99] Steele A.C., Mullins L.L., Mullins A.J., Muriel A.C. (2015). Psychosocial Interventions and Therapeutic Support as a Standard of Care in Pediatric Oncology. Pediatr. Blood Cancer.

[100] Gerhardt C.A., Lehmann V., Long K.A., Alderfer M.A. (2015). Supporting Siblings as a Standard of Care in Pediatric Oncology Psychosocial Standard of Care. Pediatr. Blood Cancer.

[101] Weaver M.S., Heinze K.E., Kelly K.P., Wiener L., Casey R.L., Bell C.J., Wolfe J., Garee A.M., Watson A., Hinds P.S. (2015). Palliative Care as a Standard of Care in Pediatric Oncology. Pediatr. Blood Cancer.

[102] Lichtenthal W.G., Sweeney C.R., Roberts K.E., Corner G.W., Donovan L.A., Prigerson H.G., Wiener L. (2015). Bereavement Follow-Up after the Death of a Child as a Standard of Care in Pediatric Oncology. Pediatr Ann..

